# Current Status of Patent Ductus Arteriosus Stenting: An International Practice Based Study

**DOI:** 10.1007/s00246-026-04236-w

**Published:** 2026-03-27

**Authors:** Colin J. McMahon, Danica Peterson, Howaida El Said

**Affiliations:** 1 Department of Paediatric Cardiology, Children’s Health Ireland, Crumlin, Dublin 12, Ireland; 2 School of Medicine, University College Dublin, Dublin, Ireland; 3 Rady Children’s Hospital, San Diego, CA USA

**Keywords:** Patent ductus arteriosus, Ductal stent, Congenital heart disease, Hypoplastic left heart syndrome, Norwood, Hybrid Stage I, Antiplatelet therapy, ECMO

## Abstract

Patent ductus arteriosus (PDA) stenting has become an accepted alternative to the modified Blalock–Taussig–Thomas shunt (mBTTS) for duct-dependent pulmonary blood flow (DDPBF). However, several key aspects of practice, including prostaglandin (PGE) titration, antiplatelet therapy, access strategy, back-up planning, and hypoplastic left heart syndrome (HLHS) palliation, remain highly variable. We conducted an international web-based survey of interventional pediatric cardiologists. Domains included PGE management, antiplatelet therapy, platelet function testing, stent-length selection, ECMO and surgical back-up, carotid access, complication profile. A total of 138 respondents participated. PGE strategies varied widely: in single-source pulmonary blood flow, 40% used saturation-guided titration, and 29% routinely discontinued PGE; in dual-source circulation, 40% always discontinued PGE and 34% used titration-based strategies. Aspirin monotherapy (46%) and dual antiplatelet therapy (45%) were equally used; clopidogrel doses typically approximated 0.2 mg/kg/day. Platelet function testing was rarely used (19%). Stent-length selection relied on straight-line measurement in 41% and tortuous central-line measurement in 39%. ECMO support was never available in 35% of centers, selectively available in 23%, and always available in 21%. A surgeon was routinely on standby in 48%. Carotid access was used percutaneously by 45% and surgically by 24%. Complications encountered by operators at any stage during their performing these procedures included ductal spasm (87%), thrombosis (51%), LPA “shut-down” (36%), and ductal dissection (33%). Contemporary PDA-stent practice varies markedly across institutions, particularly regarding PGE management, antiplatelet therapy, technical strategy, and back-up planning. HLHS and interstage practices show similar heterogeneity. These findings support the need for multicenter registries and consensus recommendations.

## Introduction

Over two decades, PDA stenting has evolved into a mainstream alternative to mBTTS for neonates with DDPBF [1–4]. Early single-center experience demonstrated that stenting can provide stable pulmonary blood flow, promote balanced pulmonary artery growth, and enable progression to definitive repair across a wide anatomic spectrum [1–8].

Recent multicenter comparisons and meta-analyses show that PDA stenting yields similar or improved survival versus surgical shunts in selected populations, with shorter intensive-care stays and comparable pulmonary artery growth, albeit with higher reintervention rates [9–16]. Advances in ductal morphology classification, carotid/axillary access, stent platforms, and imaging have made previously unfavorable ducts, vertical, tortuous, complex, amenable to stenting [5–8, 11–13, 17–22].

PDA stenting is integral to hybrid Stage I palliation for HLHS and related lesions, replacing the Norwood’s systemic-to-pulmonary shunt with bilateral pulmonary artery banding and ductal stenting [23–27]. Concurrently, structured home-monitoring programs have improved interstage survival [28–30].

Despite this progress, fundamental aspects of PDA-stent practice remain empiric. Evidence guiding PGE titration, ideal antiplatelet regimens, stent-length methodology, and resource requirements (ECMO, surgical standby) is limited. HLHS management, Norwood versus hybrid, and post-Norwood interstage strategies also vary widely.

We therefore conducted an international survey to characterize current PDA-stent practices, with emphasis on PGE management, antiplatelet therapy, access strategy, complications, HLHS approach, and interstage care.

## Materials and Methods

### Study Design and Participants

A cross-sectional web-based survey was distributed to interventional pediatric cardiologists who attended a workshop/meeting on PDA stent management at Rady Children’s Hospital, between September and November 2025 via professional mailing lists, networks, and society channels. Participation was voluntary and anonymous unless respondents elected to provide identifying details.

### Survey Instrument

The survey included structured multiple-choice and optional free-text questions covering:

- PGE management (single- and dual-source pulmonary blood flow)
- Antiplatelet regimen and platelet function testing
- Stent-length strategy
- ECMO availability and surgical back-up
- Carotid access
- Complications
- Preferred HLHS strategy
- Post-Norwood interstage practices

### Data Analysis

Responses were analyzed descriptively. Categorical variables are reported as counts and percentages. No hypothesis testing was performed.

## Results

A total of 138 interventional cardiologists from geographically diverse centers participated in the survey, representing North America, Europe, Asia, the Middle East, and Latin America. Respondents included operators from both large, high-volume congenital cardiac centers as well as smaller programs, as reflected in the free-text institutional descriptions. The distribution of responses across all major domains is summarized below.

### PGE Management

#### Single-Source Pulmonary Blood Flow

**Fig. 1:**
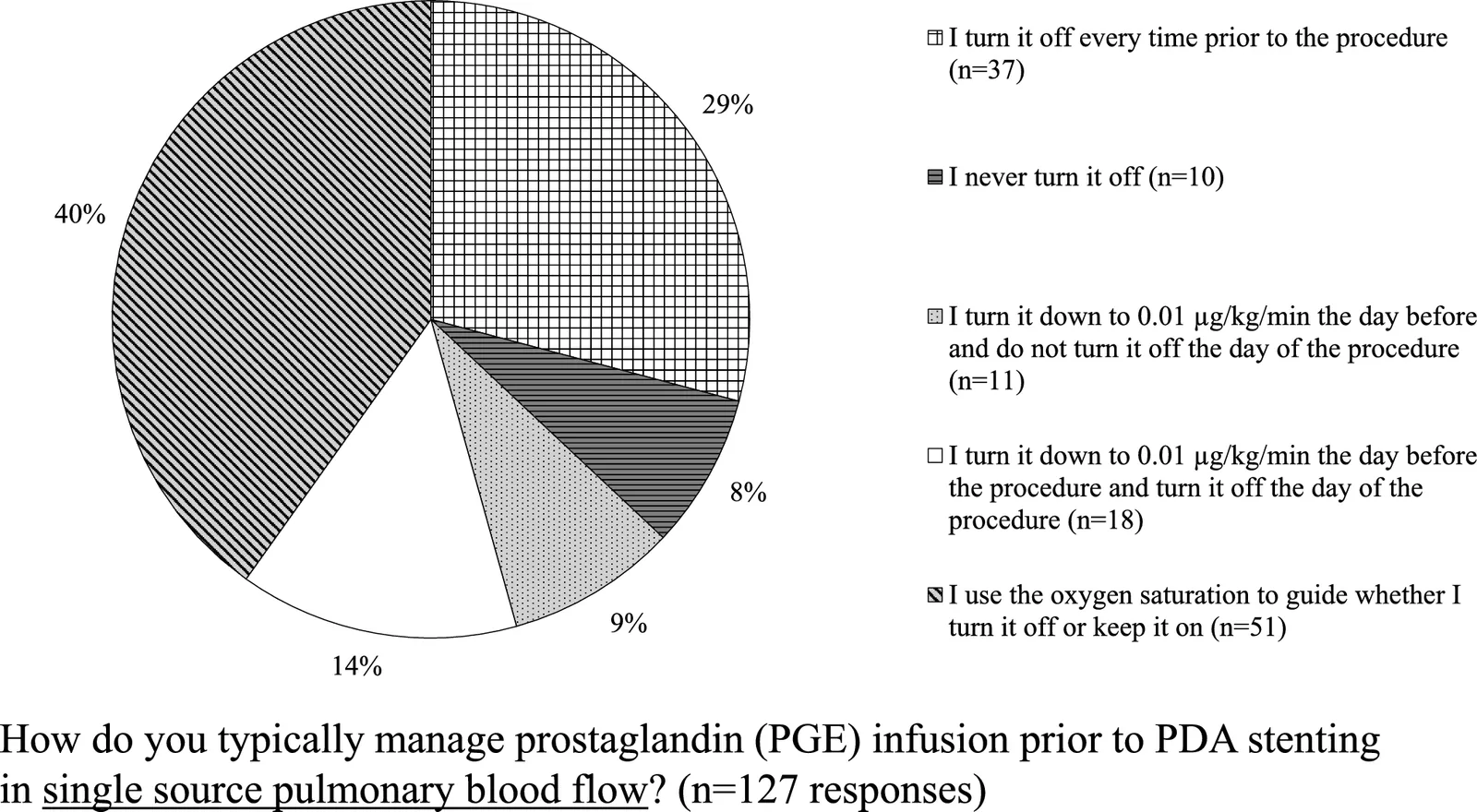
Prostaglandin management prior to patent ductus arteriosus stenting in single source pulmonary blood flow

Among 127 respondents, PGE practice in single-source pulmonary blood flow showed substantial variation (Fig. 1). The most common approach (40%) was saturation- or clinically guided titration, wherein operators incrementally down-titrated PGE while monitoring oxygen saturation, perfusion, or hemodynamic stability. A sizable minority (29%) routinely discontinued PGE before the procedure regardless of ductal morphology or clinical stability. An additional 14% reduced PGE to very low doses (often 0.01 μg/kg/min) before stopping on the day of the procedure.

A total of 9% decreased PGE dose but intentionally maintained a low infusion until wire traversal to minimize the risk of ductal spasm or abrupt closure during catheter manipulation. A small subset (8%) never discontinued PGE prior to stenting. Free-text comments emphasized the delicate balance between avoiding ductal constriction and preventing pulmonary over-circulation, particularly in long or tortuous ducts.

### Dual-Source Pulmonary Blood Flow

**Fig. 2:**
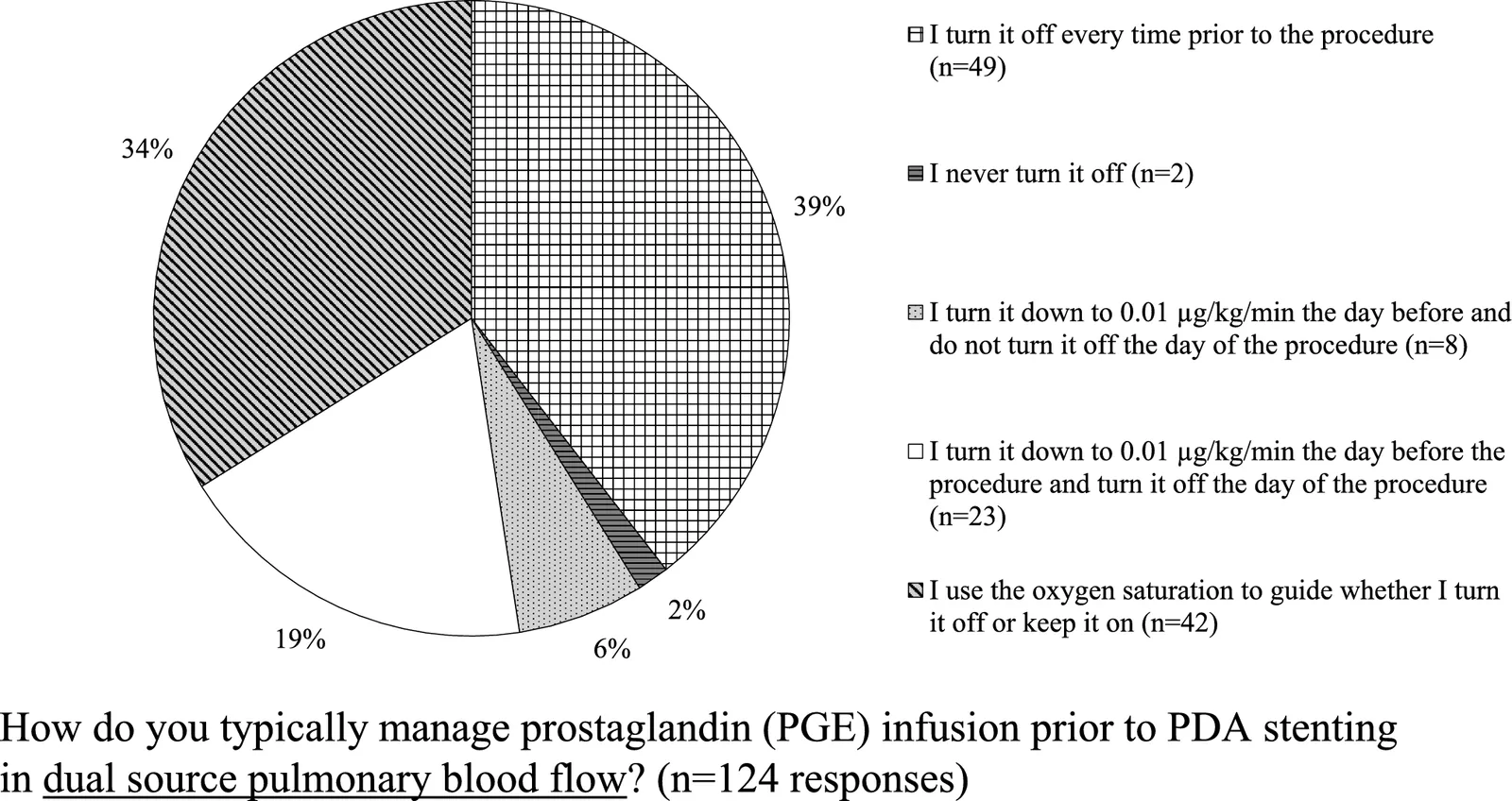
Prostaglandin management prior to patent ductus arteriosus stenting in dual source pulmonary blood flow

Among 124 respondents, PGE discontinuation was more routine in dual-source physiology (Fig. 2). Forty percent always discontinued PGE prior to catheterization, while 34% used saturation-guided titration. A staged weaning approach was reported by 19%, and 7% reduced PGE dose without stopping entirely. Only 2% never discontinued PGE in this setting. Respondents consistently noted a lower perceived risk of catastrophic ductal closure in dual-source physiology.

### Antiplatelet Therapy and Platelet Function Testing

**Fig. 3:**
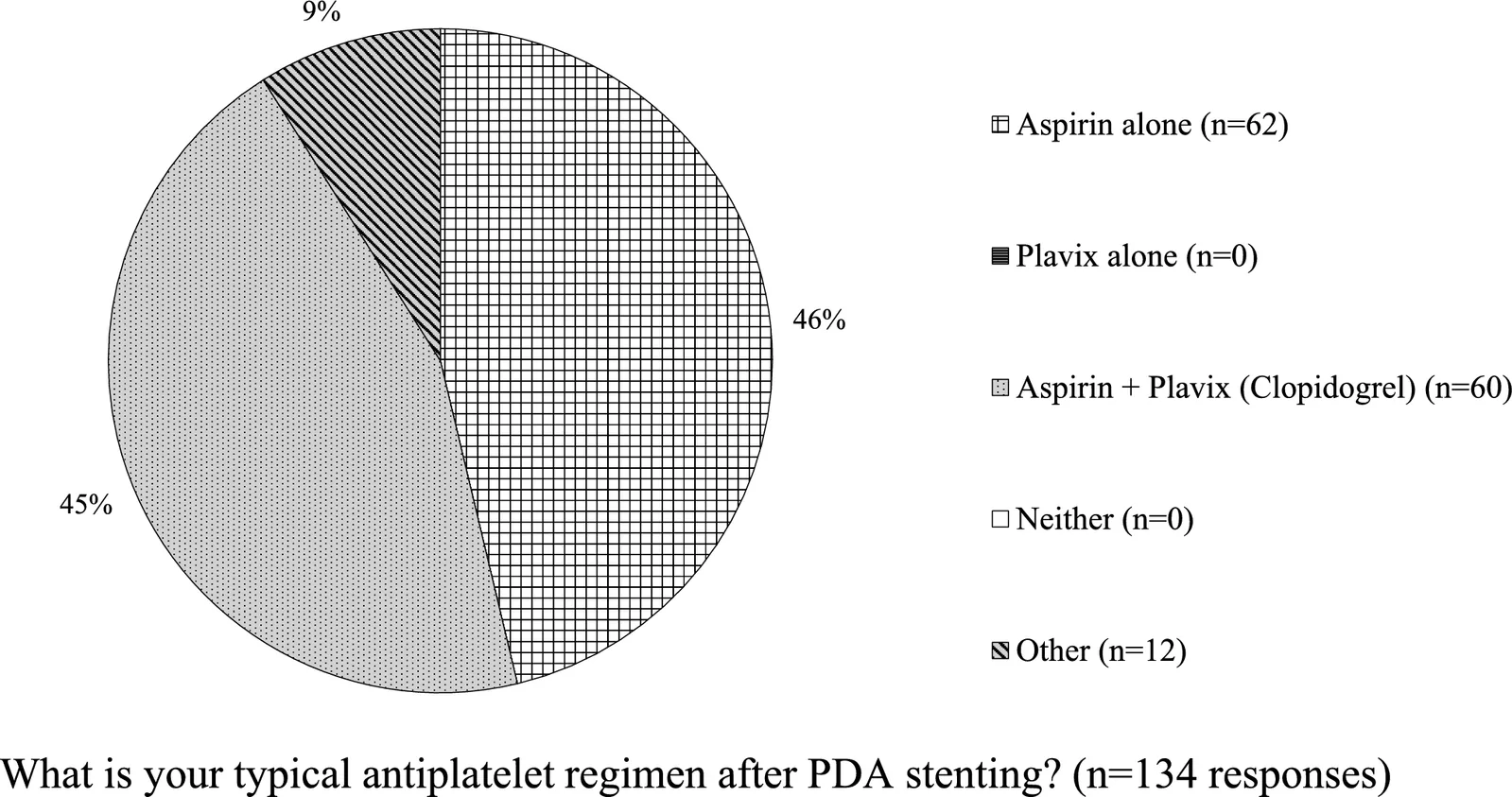
Antiplatelet therapy regimen practice variation after stenting of the patent ductus arteriosus

Antiplatelet strategies varied almost evenly between aspirin monotherapy and dual antiplatelet therapy (DAPT) (Fig. 3). Among those specifying standard regimens, 46% used aspirin alone, while 45% prescribed DAPT with aspirin plus clopidogrel. Operators opting for DAPT typically favored clopidogrel doses of approximately 0.2 mg/kg/day, consistent with pediatric pharmacodynamic evidence [17]. Only a minority used loading doses or higher maintenance dosing.

Platelet function testing was uncommon: only 19% routinely used VerifyNow or a comparable assay. Respondents cited several challenges, limited blood volume in neonates, cost, logistic constraints, inconsistent normative data, and uncertain clinical impact, as key barriers.

### Stent-Length Selection

**Fig. 4:**
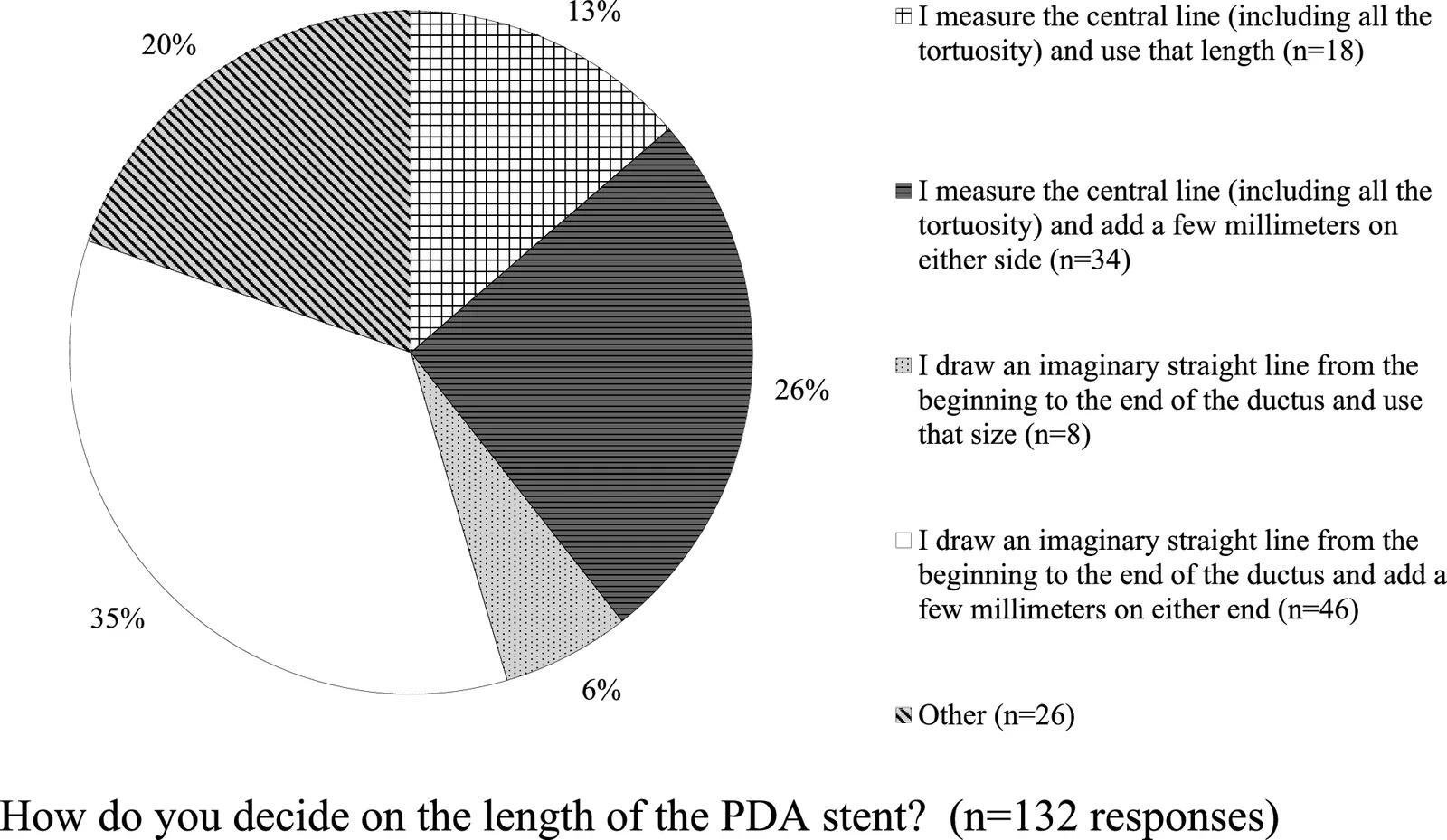
Decision on stent length in stenting patent ductus arteriosus

Of 132 respondents, stent-length planning followed two nearly equally adopted paradigms (Fig. 4). Straight-line length (aortic origin to pulmonary insertion) was used by 41%, while 39% preferred measuring the duct’s tortuous central axis to ensure full coverage. The remaining 20% relied on individualized strategies incorporating 3D reconstructions, CT angiography, pre- and post-wire angiography, or real-time angiographic adjustment. Respondents acknowledged the tension between ensuring complete coverage and avoiding protrusion into adjoining structures.

### ECMO and Surgical Back-Up

**Fig. 5:**
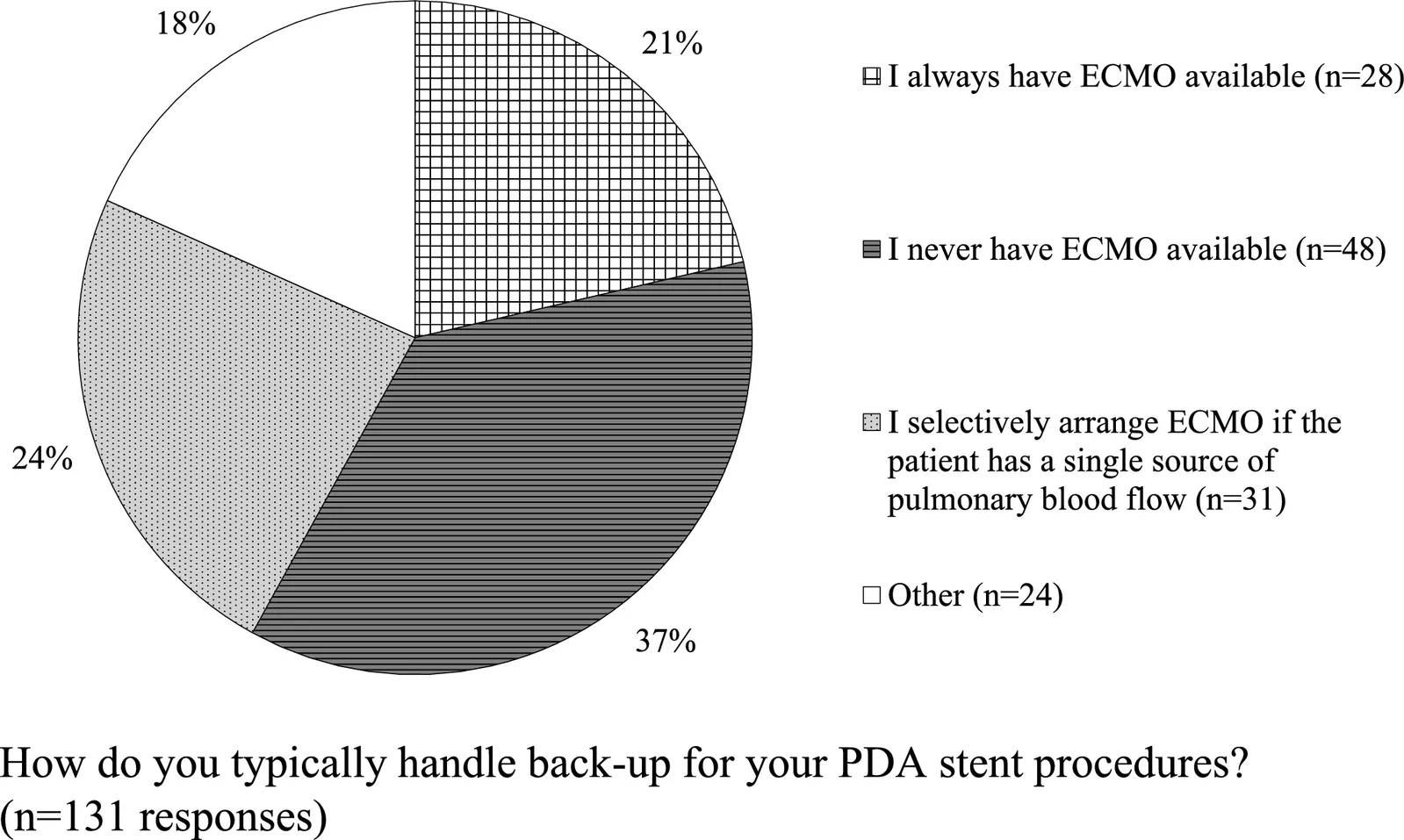
Back-up with ECMO for PDA stenting procedures

Wide variability existed in institutional back-up resources (Fig. 5). Among 131 respondents, ECMO was never available in 35% of centers, selectively available for high-risk cases in 23%, and consistently available in 21%. Some centers reported hybrid arrangements where ECMO could be mobilized but not immediately. Surgical standby was more common: 48% had a congenital surgeon routinely available during PDA stenting, while 36% arranged standby selectively for complex ductal morphology or single-source physiology. Sixteen percent reported no formal surgical standby availability.

### Carotid Access

Carotid artery access was widely adopted, with 45% using a percutaneous carotid approach and 24% performing a surgical cut-down. Thirty-one percent avoided carotid access altogether, typically citing institutional preference or comfort with femoral or axillary access. Free-text responses highlighted the perceived benefits of carotid access for vertical or sharply angulated ducts, with several respondents referencing improved wire alignment and procedural stability.

### Complications

**Fig. 6:**
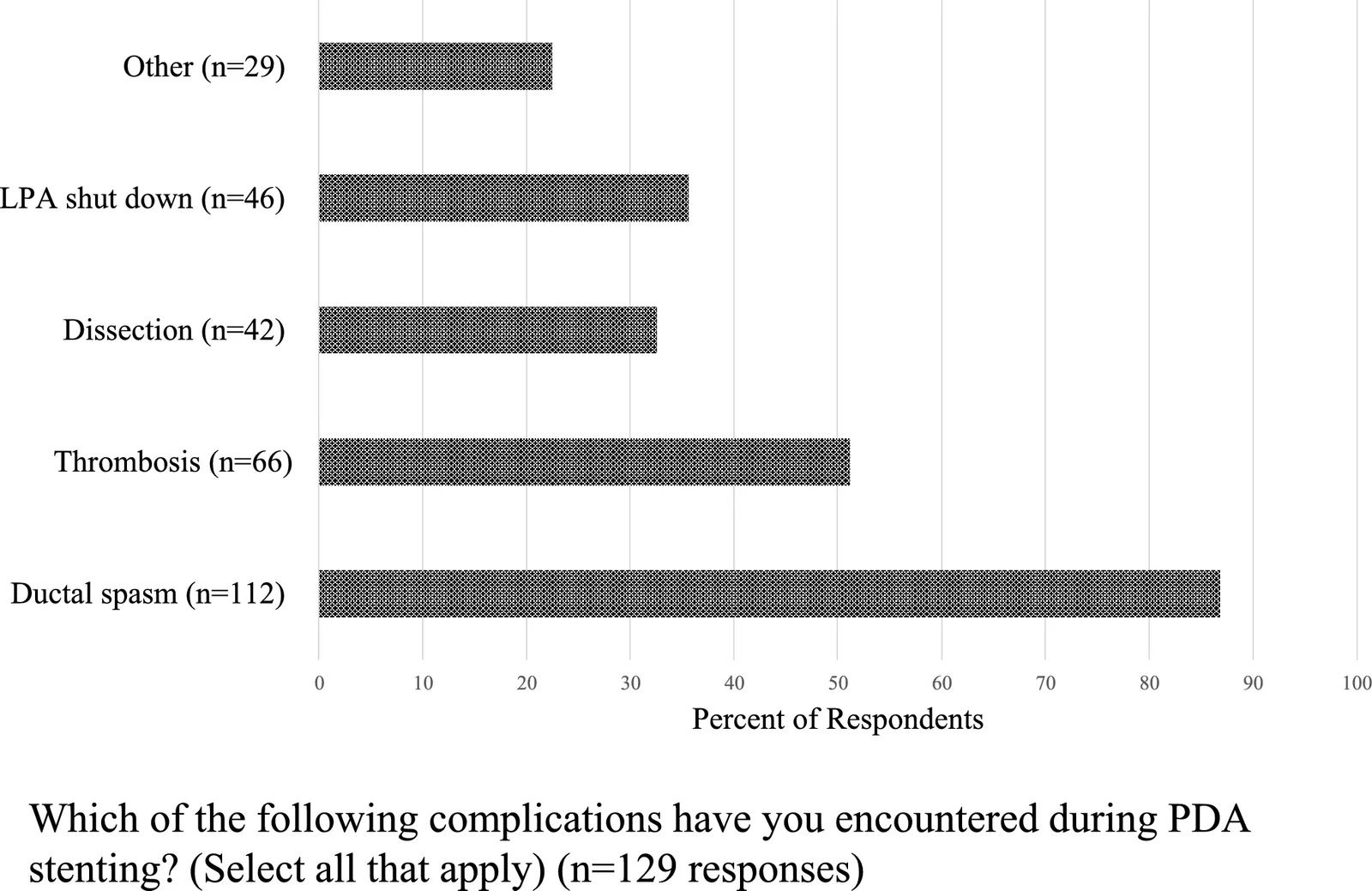
Complications encountered by operators at any stage during their experience of performing these procedures

Complications were reported in a multi-select format by 129 respondents (Fig. 6). Complications encountered by operators at any stage during their experience of performing these procedures included ductal spasm (87%), thrombosis (51%), LPA “shut-down” (36%), and ductal dissection (33%). Other complications, including stent migration, branch pulmonary artery jailing, access-site thrombosis, and rare catastrophic events such as coronary compression, were reported less frequently. Several respondents noted a decrease in complication rates over time with growing institutional experience.

### HLHS Strategy

Among 105 respondents, the majority (69%) favored a conventional Norwood procedure for standard-risk HLHS. Sixteen percent used initial pulmonary blood flow restriction (e.g., banding) followed by Norwood, whereas 15% preferred a primary hybrid Stage I strategy combining ductal stenting and bilateral pulmonary artery banding. Respondents commonly reserved hybrid palliation for high-risk infants or specific anatomical substrates.

### Post-Norwood Interstage Management

Among 106 respondents, 70% discharged patients home when clinically ready, typically with structured home-monitoring programs. Twenty-three percent hospitalized only high-risk infants until Stage II, and 8% kept all post-Norwood infants inpatient until Stage II. Respondents noted that socioeconomic context and regional resource variability influenced interstage discharge decisions.

## Discussion

This international survey highlights the considerable heterogeneity in contemporary PDA-stent practice and associated HLHS management across diverse cardiac centers. Although PDA stenting has become an increasingly accepted alternative to surgical systemic-to-pulmonary shunts for duct-dependent pulmonary blood flow, several fundamental aspects of peri-procedural and interstage management remain variable. The findings underscore the absence of unified, evidence-based guidelines and the need for multicenter collaboration to optimize outcomes.

One of the most striking findings is the range of PGE management strategies. In single-source pulmonary blood flow, abrupt ductal closure can be catastrophic, prompting many operators to use a cautious, clinically guided titration approach. The continued use of low-dose PGE until the wire has crossed the ductus reflects an emphasis on procedural safety in the setting of complex ductal anatomy. Despite two decades of PDA-stent experience, there remains remarkably little published guidance to inform PGE titration [1–8, 17, 21]. The variability seen in this survey highlights an unmet need for prospective research comparing standardized titration strategies and assessing associations with spasm, access-site selection, procedural times, and early outcomes.

Antiplatelet therapy is another area lacking consensus. Although nearly half of respondents use dual antiplatelet therapy with aspirin and clopidogrel, the clinical evidence supporting this practice remains sparse [17, 23]. Data from small pediatric cohorts suggest target platelet inhibition with the commonly used 0.2 mg/kg/day clopidogrel dose, but the true impact of DAPT on stent patency and thrombosis prevention remains unclear, particularly given the bleeding risk in neonates. Similarly, platelet function testing remains infrequent, reflecting its uncertain benefit, practical limitations, and financial burden. The field would benefit from multicenter prospective registries examining thrombosis, bleeding rates, and reintervention according to antiplatelet regimen and platelet reactivity profiles.

Technical aspects of PDA-stent implantation continue to evolve, and this survey underscores ongoing variability in stent-length selection. Straight-line measurements risk underestimating the length needed to fully cover a tortuous duct, whereas central-line measurements risk excessive protrusion into adjacent vasculature [1–3, 5–8, 11–13]. The growing adoption of advanced imaging, including 3D angiography and CT-based modeling, reflects operators’ efforts to better characterize ductal anatomy. However, such technology is not universally available. The field may benefit from consensus statements delineating when each strategy may be optimal, ideally supported by multicenter outcome comparisons.

The survey also reveals considerable differences in institutional readiness for high-risk PDA stenting. ECMO availability ranged from never to always available, and surgical back-up also varied significantly. Centers without ECMO or surgical standby tended to restrict PDA stenting to anatomies deemed low risk, reflecting prudent resource-aware decision-making. Although PDA stenting generally demonstrates outcomes comparable or superior to surgical shunting in selected patients [9–16], these results may not be generalizable to settings with limited back-up resources. As PDA stenting continues to expand globally, scalable frameworks for safe implementation—including minimum resource recommendations—may be warranted.

Carotid access has emerged as an important strategy for vertical or tortuous ducts and was used percutaneously by nearly half of all respondents. Recent data reassure operators about neurologic and vascular safety when carotid access is performed in experienced centers [7, 8, 19]. Yet the long-term effects on carotid patency remain poorly defined. The widespread adoption of carotid access reported here suggests growing comfort among operators, but highlights the need for dedicated long-term vascular follow-up studies.

Regarding HLHS palliation, the majority of respondents favored the conventional Norwood approach for standard-risk infants. This aligns with contemporary multicenter analyses suggesting that hybrid palliation, while valuable for high-risk infants or specific institutional models, may confer increased procedural burden and reintervention without clear long-term survival benefit in standard-risk populations [24–28]. Nevertheless, the 15% adoption rate for primary hybrid Stage I reflects its continued role in select settings, especially where early surgical risk is prohibitive. As hybrid techniques continue to evolve, outcomes across different institutional models should be compared to define optimal patient selection.

Interstage management after Norwood remains another area of variability. While 70% of respondents discharge patients home with monitoring, the intensity and structure of these programs vary widely. Evidence clearly demonstrates that structured home-surveillance programs reduce interstage mortality [29–31]. The finding that some centers continue to hospitalize all or nearly all post-Norwood infants suggests persisting concerns regarding resource availability, family reliability, and outpatient monitoring infrastructure. Global harmonization of interstage monitoring protocols may therefore represent one of the most impactful next steps in improving survival.

Overall, this survey highlights an important tension in contemporary PDA-stent practice: while accumulating evidence supports PDA stenting as a safe and effective alternative to surgical shunting, significant practice variation persists across virtually all domains. Establishing best practices will require coordinated multicenter efforts, robust prospective registries, and consensus-based guidance documents addressing peri-procedural management, access strategy, stent selection, and interstage surveillance. Until such recommendations are developed, operators should emphasize multidisciplinary collaboration, careful patient selection, and institution-specific safety protocols to mitigate risk and improve outcomes.

## Data Availability

Data is available on request.
